# Supplementary material for: Health burden and economic costs of smoking in Chile: The potential impact of increasing cigarettes prices
Source: PLoS One. 2020 Aug 28;15(8):e0237967. doi: 10.1371/journal.pone.0237967 (PMC7454964; doi:10.1371/journal.pone.0237967)
Supplement: S1 Table — (DOCX) [file pone.0237967.s001.docx]

**S1 Table. Smoking prevalence and total population by single age and sex in Chile, 2017**

| **Age** | **Males prevalence** | | **Females prevalence** | | **Population** | |
| --- | --- | --- | --- | --- | --- | --- |
|  | ***Smokers*** | ***Ex- smokers*** | ***Smokers*** | ***Ex- smokers*** | ***Male*** | ***Female*** |
| 35 | 49.51% | 23.59% | 35.72% | 23.61% | 130,841 | 133,618 |
| 36 | 49.51% | 23.59% | 35.72% | 23.61% | 121,700 | 124,050 |
| 37 | 49.51% | 23.59% | 35.72% | 23.61% | 119,075 | 122,340 |
| 38 | 49.51% | 23.59% | 35.72% | 23.61% | 113,110 | 117,330 |
| 39 | 49.51% | 23.59% | 35.72% | 23.61% | 110,882 | 114,831 |
| 40 | 49.51% | 23.59% | 35.72% | 23.61% | 112,362 | 116,615 |
| 41 | 49.51% | 23.59% | 35.72% | 23.61% | 115,482 | 120,128 |
| 42 | 49.51% | 23.59% | 35.72% | 23.61% | 118,576 | 124,160 |
| 43 | 49.51% | 23.59% | 35.72% | 23.61% | 121,374 | 125,980 |
| 44 | 49.51% | 23.59% | 35.72% | 23.61% | 118,880 | 124,946 |
| 45 | 31.30% | 36.78% | 29.84% | 23.67% | 116,190 | 122,949 |
| 46 | 31.30% | 36.78% | 29.84% | 23.67% | 112,420 | 119,016 |
| 47 | 31.30% | 36.78% | 29.84% | 23.67% | 109,315 | 116,169 |
| 48 | 31.30% | 36.78% | 29.84% | 23.67% | 110,844 | 118,665 |
| 49 | 31.30% | 36.78% | 29.84% | 23.67% | 113,714 | 121,481 |
| 50 | 31.30% | 36.78% | 29.84% | 23.67% | 114,143 | 122,355 |
| 51 | 31.30% | 36.78% | 29.84% | 23.67% | 114,178 | 122,487 |
| 52 | 31.30% | 36.78% | 29.84% | 23.67% | 115,044 | 124,505 |
| 53 | 31.30% | 36.78% | 29.84% | 23.67% | 112,286 | 121,883 |
| 54 | 31.30% | 36.78% | 29.84% | 23.67% | 114,201 | 123,872 |
| 55 | 31.30% | 36.78% | 29.84% | 23.67% | 107,543 | 117,563 |
| 56 | 31.30% | 36.78% | 29.84% | 23.67% | 104,214 | 114,390 |
| 57 | 31.30% | 36.78% | 29.84% | 23.67% | 98,460 | 108,568 |
| 58 | 31.30% | 36.78% | 29.84% | 23.67% | 95,766 | 105,624 |
| 59 | 31.30% | 36.78% | 29.84% | 23.67% | 93,423 | 102,228 |
| 60 | 31.30% | 36.78% | 29.84% | 23.67% | 90,565 | 99,591 |
| 61 | 31.30% | 36.78% | 29.84% | 23.67% | 83,298 | 93,064 |
| 62 | 31.30% | 36.78% | 29.84% | 23.67% | 78,101 | 87,478 |
| 63 | 31.30% | 36.78% | 29.84% | 23.67% | 76,312 | 86,163 |
| 64 | 31.30% | 36.78% | 29.84% | 23.67% | 71,286 | 81,057 |
| 65 | 15.69% | 45.68% | 9.05% | 32.65% | 66,949 | 75,864 |
| 66 | 15.69% | 45.68% | 9.05% | 32.65% | 62,630 | 71,888 |
| 67 | 15.69% | 45.68% | 9.05% | 32.65% | 60,688 | 70,068 |
| 68 | 15.69% | 45.68% | 9.05% | 32.65% | 57,424 | 66,908 |
| 69 | 15.69% | 45.68% | 9.05% | 32.65% | 55,568 | 65,015 |
| 70 | 15.69% | 45.68% | 9.05% | 32.65% | 52,721 | 62,724 |
| 71 | 15.69% | 45.68% | 9.05% | 32.65% | 49,543 | 59,677 |
| 72 | 15.69% | 45.68% | 9.05% | 32.65% | 46,966 | 56,572 |
| 73 | 15.69% | 45.68% | 9.05% | 32.65% | 42,855 | 52,891 |
| 74 | 15.69% | 45.68% | 9.05% | 32.65% | 40,824 | 51,136 |
| 75 | 15.69% | 45.68% | 9.05% | 32.65% | 37,434 | 47,926 |
| 76 | 15.69% | 45.68% | 9.05% | 32.65% | 34,526 | 45,515 |
| 77 | 15.69% | 45.68% | 9.05% | 32.65% | 31,387 | 42,617 |
| 78 | 15.69% | 45.68% | 9.05% | 32.65% | 27,321 | 37,281 |
| 79 | 15.69% | 45.68% | 9.05% | 32.65% | 24,858 | 34,724 |
| 80 | 15.69% | 45.68% | 9.05% | 32.65% | 23,225 | 33,152 |
| 81 | 15.69% | 45.68% | 9.05% | 32.65% | 20,772 | 31,067 |
| 82 | 15.69% | 45.68% | 9.05% | 32.65% | 18,803 | 28,733 |
| 83 | 15.69% | 45.68% | 9.05% | 32.65% | 16,650 | 26,148 |
| 84 | 15.69% | 45.68% | 9.05% | 32.65% | 15,546 | 25,350 |
| 85 | 15.69% | 45.68% | 9.05% | 32.65% | 13,840 | 23,079 |
| 86 | 15.69% | 45.68% | 9.05% | 32.65% | 12,813 | 23,124 |
| 87 | 15.69% | 45.68% | 9.05% | 32.65% | 10,715 | 20,171 |
| 88 | 15.69% | 45.68% | 9.05% | 32.65% | 8,594 | 16,920 |
| 89 | 15.69% | 45.68% | 9.05% | 32.65% | 7,507 | 15,038 |
| 90 | 15.69% | 45.68% | 9.05% | 32.65% | 5,644 | 11,752 |
| 91 | 15.69% | 45.68% | 9.05% | 32.65% | 4,153 | 9,408 |
| 92 | 15.69% | 45.68% | 9.05% | 32.65% | 3,566 | 8,276 |
| 93 | 15.69% | 45.68% | 9.05% | 32.65% | 2,612 | 6,175 |
| 94 | 15.69% | 45.68% | 9.05% | 32.65% | 2,054 | 5,243 |
| 95 | 15.69% | 45.68% | 9.05% | 32.65% | 1,463 | 3,760 |
| 96 | 15.69% | 45.68% | 9.05% | 32.65% | 1,100 | 3,170 |
| 97 | 15.69% | 45.68% | 9.05% | 32.65% | 744 | 2,091 |
| 98 | 15.69% | 45.68% | 9.05% | 32.65% | 506 | 1,493 |
| 99 | 15.69% | 45.68% | 9.05% | 32.65% | 375 | 1,154 |
| 100 | 15.69% | 45.68% | 9.05% | 32.65% | 375 | 1,154 |
