# Supplementary material for: Health burden and economic costs of smoking in Chile: The potential impact of increasing cigarettes prices
Source: PLoS One. 2020 Aug 28;15(8):e0237967. doi: 10.1371/journal.pone.0237967 (PMC7454964; doi:10.1371/journal.pone.0237967)
Supplement: S2 Table — (DOCX) [file pone.0237967.s002.docx]

**S2 Table. Relative risks of mortality (RR) for smokers and ex-smokers for each tobacco-related condition, by sex (in reference to never-smokers)**

| **Tobacco-related condition** | **Classification of diseases ICD- 10** | **RR Men** | | **RR Women** | |
| --- | --- | --- | --- | --- | --- |
|  |  | **Smokers <65 / ≥65 years** | **Ex-Smokers  <65 / ≥65 years** | **Smokers <65 / ≥65 years** | **Ex-Smokers  <65 / ≥65 years** |
| Acute myocardial infarction | I210-I229 | 2.80 / 1.51 | 1.64 / 1.21 | 3.08 / 1.60 | 1.32 / 1.20 |
| Unstable angina | I200-I209 | 2.80 / 1.51 | 1.64 / 1.21 | 3.08 / 1.60 | 1.32 / 1.20 |
| Other cardiovascular deaths | See detail ** | 1.78 / 1.78 | 1.22 / 1.22 | 1.49 / 1.49 | 1.14 / 1.14 |
| Stroke | I60; I61; I63; I64; I620: I621 I629; I678 I679; I690 I691 I692 I693 I694 I698 | 3.27 / 1.63 | 1.04 / 1.04 | 4.00 / 1.49 | 1.30 / 1.03 |
| Lung cancer | C330-C349 | 23.26 / 23.26 | 8.70 / 8.70 | 12.69 / 12.69 | 4.53 / 4.53 |
| Pneumonia/influenza | J100-J189 | 1.75 / 1.75 | 1.36 / 1.36 | 2.17/2.17 | 1.10 / 1.10 |
| Bronchitis and emphysema | J400-J439 | 10.58 / 10.58 | 6.80 / 6.80 | 13.08 / 13.08 | 6.78 / 6.78 |
| Airway obstruction | J44X | 1.26 / 1.26 | 1.15 / 1.15 | 1.26 / 1.26 | 1.15 / 1.15 |
| Mouth and pharyngeal cancer | C000 a C009; C140; C142 C148 | 10.89 / 10.89 | 3.40 / 3.40 | 5.08 / 5.08 | 2.29 / 2.29 |
| Esophageal cancer | C150-C159 | 6.76 / 6.76 | 4.46 / 4.46 | 7.75 / 7.75 | 2.79 / 2.79 |
| Stomach cancer | C160-C169 | 1.96 / 1.96 | 1.47 / 1.47 | 1.36 / 1.36 | 1.32 / 1.32 |
| Pancreatic cancer | C250-C259 | 2.31 / 2.31 | 1.15 / 1.15 | 2.25 / 2.25 | 1.55 / 1.55 |
| Kidney cancer | C64X-C65X | 2.72 / 2.72 | 1.73 / 1.73 | 1.29 / 1.29 | 1.05 / 1.05 |
| Laryngeal cancer | C320-C329 | 14.6 / 14.6 | 6.34 / 6.34 | 13.02 / 13.02 | 5.16 / 5.16 |
| Leukemia | C920 | 1.86 / 1.86 | 1.33 / 1.33 | 1.38 / 1.38 | 1.13 / 1.13 |
| Bladder cancer | C670-C679 | 3.27 / 3.27 | 2.09 / 2.09 | 2.22 / 2.22 | 1.89 / 1.89 |
| Cervical cancer | C530-C539 | 1 / 1 | 1 / 1 | 1.59 / 1.59 | 1.14 / 1.14 |
| ***I00; I010-I012; I018-I020; I029; I050-I052; I058; I062; I068-I072; I078-I083; I088-I092; I098-I099; I110; I119; I260; I269-I272; I278 I281; I288; I289; I300; I301; I308-I313; I318-I319; I320; I321; I328; I3310; I339-I342; I348-I352; I358-I362; I368-I372; I378; I379; I38X; I390-I394; I398; I400; I401; I408; I409; I410-I412; I418; I420-I429; I430-I432; I438; I440-I447; I450-I456; I458-I461; I469-I472; I479; I48X; I490-I495; I498-I501; I509-I519; I059.I060-1; I700-I702; I708; I709; I710-I719; I720-I729. I730-I739. I740-I749. I770-I779; I780-I789.* | | | | | |
