# Supplementary material for: Health burden and economic costs of smoking in Chile: The potential impact of increasing cigarettes prices
Source: PLoS One. 2020 Aug 28;15(8):e0237967. doi: 10.1371/journal.pone.0237967 (PMC7454964; doi:10.1371/journal.pone.0237967)
Supplement: Attachment — Submitted filename: rebuttal letter_PONE-D-20-00908R1.docx [file pone.0237967.s004.docx]

Santiago, August 4^th^, 2020

PONE-D-20-00908R1
**Health burden and economic costs of smoking in Chile: The potential impact of increasing cigarettes prices**

Dear Mr. Glantz,

Academic Editor
PLOS ONE

Thank you very much for your email sent on June the 22^nd^ regarding our paper resubmission. We are very grateful for the positive feedback received from the reviewers on how we addressed the previous comments.

We thank Reviewer 1 for the various additional suggestions to further improving the manuscript. The majority of these were adopted literally, while a few were treated differently, due to some specific implications, that we discuss in the manuscript and below in the present rebuttal letter, which addresses the comments made one by one.

We look forward to hearing from you hopefully, with a positive decision.

With best regards,

Marianela Castillo-Riquelme

Answer to the each of the editorial suggestions from Reviewer 1

Reviewer #1: The authors did a comprehensive response to reviewers’ comments, and the revision was well-done. I have no more major comments but have many editorial suggestions to further improve this manuscript.

1. In response to Review 2’s comment #2, the authors have replaced the term “tobacco-attributable burden” with “smoking-attributable burden” in the Results and Discussion sections given that this study focused on smoking. Given the same reason, I suggest revising the title by replacing the term “tobacco” with “smoking”.

Response: Thank you. This was implemented

2. In the Abstract, line 23 shows “ …. 33.4% prevalence of daily smokers”. This number was not reported elsewhere in the paper. Is this a typo? Given that the prevalence of current smokers in 2017 was 33.3%, how would it possible that the prevalence of daily smokers is greater than 33.3%?

Response: This was corrected. The percentage corresponds to current smokers (33.3%).

3. In the Abstract, line 28: “46 people die daily from tobacco-related diseases caused by smoking”. This finding was not shown in the Results section of the main text. I suggest replacing it with “16,472 deaths were attributable to smoking in 2017”.

Response: This was implemented

4. In the Abstract, the sentence shown on lines 38-39, “In Chile, the tobacco tax collection does not fully cover …”, should belong to the Results rather than the Conclusion. Moreover, the last sentence, “Additionally, …. Smoking-cessation …”, does not seem to be related to the findings reported above.

Response: Regarding the first sentence “In Chile, the tobacco tax collection does not fully cover …” we moved it to the results section as suggested by the reviewer.

Regarding the second point, we removed the sentence from the abstract.

We agree that there is no a direct link to the main analyses of our study. However, we believe that it is important to mention, that for Chile it is crucial to advance in all possible ways to reduce the burden of smoking. In addition to taxes, the country is lagging behind in terms of smoking cessation programs, which for ethical reasons should go hand in hand with other more restrictive measures, such as raising taxes, adopting plain packaging, and imposing smoke-free environments. Therefore, the lack of smoking cessation interventions was addressed in the introduction and in the discussion section.

5. Line 94: “… an increased in relation to”. Is “increased” a typo of “increase”?

Response: corrected – line 94

6. Line 171: “… some ex-smokers become non-smokers”. What do “non-smokers” mean in this sentence? In the response letter, the authors indicated that they have accepted the reviewer’s suggestion to change the term “non-smokers” to “never-smokers”. Does it imply that “non-smokers” referred in this sentence mean “never-smokers”?

Answer: Yes, it actually means never-smokers. We have re-written this sentence as:

“… while only in the third scenario do some ex-smokers adopt a risk similar to that of never-smokers”. line 171.

7. In line 188, the meaning of “related regulation” needs to explained.

Response: US has been implementing more strict smoking regulations for longer time (as compared to Chile), therefore indirect costs (taken in reference to US costs) are likely to be conservative when applied to Chile.

We modified the sentence, adding:

“…since the US has been implementing smoking regulation long before Chile”. Now in lines 188-189

8. In Table 1: “relative risks for smokers, ex-smokers, …” Add “of mortality” after “relative risks”.

Response: implemented

9. In Table 1, one of the cell in the last column says “See Table 2”. It should be corrected as “See Table 4”.

Response: corrected

10. In Table 1, “Tobacco price elasticity of demand [- 0.45]” would be better changed to “Price elasticity of cigarette demand [- 0.45]”.

Response: implemented

11. Lines 202-203: “In Chile, nearly 75% of the population is affiliated to the national health fund (FONASA) while around 15% is affiliated to the ISAPRES (private insurers).” Does this sentence suggest that 90% of the population is covered by public or private insurance, and 10% is uninsured?

Response: The remaining 10% comprises 1) other financial health care mechanisms, such as the Armed Forces and Police Department systems, 2) independent workers / professionals who cover their health expenses out-of-pocket, and 3) uninsured people (who is minimal).

We believe that this full explanation might not be necessary. However, we added a brief explanation, as follows:

“The remaining 10% comprises other institutional arrangements for healthcare, including a low proportion corresponding to the uninsured population" lines 205-207

12. Lines 257-262 contain the most important results from this study, namely, the estimated smoking-attributable health burden and economic costs of smoking. However, these estimates were not presented clearly enough and the distinction between “smoking-attributable” and “smoking caused diseases” still seems ambiguous. I suggest four editorial changes.
a. Line 256. Change the section title to “Mortality, morbidity, and costs of smoking”.

Response: implemented – line 258

b. Lines 257-258. Change the sentence from “… around 16,742 people die annually due to pathologies caused by smoking, a number that represents around 16.3% of all deaths to “annually there are 16,742 deaths attributable to smoking, a number that represents around 39% of tobacco-related deaths (43,322) and 16.3% of all-cased deaths (xxx,xxx)“.

Response: this recommendation was implemented, but we prefer not to add the total annual deaths at the end of the sentence. This is because, as our values are determined through modelling, absolute estimates do not always match the exact observed value. For total deaths, the model estimated slightly less (-0.031) deaths than the officially reported in Chile in 2017.

The new paragraph reads:

"In Chile, we estimate 16,742 deaths attributable to smoking annually, a figure that represents around 39% of deaths from smoking-related diseases (43,322) and about 16% of all cases of death" lines 259-260

c. Lines 259-260. Change the sentence from “180,000 events are expected each year, of which 47% are attributable to tobacco consumption“ to “180,000 events are expected each year, of which 85,000 (47%) are attributable to cigarette consumption“.

Response: this was implemented – lines 261-262

d. Lines 261. Change the sentence from “U$D 3.4 billion, of which 52% corresponds to smoking-attributable treatment costs” to “U$D 3.4 billion, of which U$D 1.8 billion (52%) are smoking-attributable treatment costs”.

Response: implemented – lines 263 -264

13. In Table 5, the last row’s label should not contain “tobacco attributable“. In other words, it should be just “Total”. For the “Smoking-attributable events” columns, the header, “%”, should be renamed as “% row”. Also, it would be helpful to add one more column to show the column percentage.

Response: implemented

14. Line 188. Change “first cause” to “top cause”.

Response: implemented - line 271

15. Line 281. Should “58%” be corrected as “69%” according to Table 6?

Response: yes, this was corrected - line 284

16. Line 308. Should “12,302 cases of AMI…” be corrected as “11,386 cases of AMI…” according to Table 5?

Response: corrected, and cases for stroke were added – line 310

17. Line 310. Add “disability-adjusted “ before “life”.

Response: added – line 312

18. Line 311. The number cited in the sentence “...spends around U$D 1.15 billion per year in direct costs of care of smoking..“ is incorrect.

Response: corrected to 1.8 – line 313

19. Line 312. Is “8.3%” a typo of “8.1%”?

Response: yes, this was corrected – line 314

20. Lines 320-321. It is not clear what specific results of this study do not differ greatly from the estimates published in 2004 [22]. Are the authors referring to smoking-attributable deaths, DALYs, or costs of smoking? And, what are the estimates from reference #22?

Response: this paragraph was rewritten as follows

"Our results do not differ much from those published in 2014, when 16,532 deaths attributable to smoking were estimated, equivalent to 18.5% of the country's annual deaths. DALY, on the other hand, had been estimated at 428,588 - 3% more than the current study [22] ". lines 327 -329

We also put this paragraph a little later, to maintain a chronological logic.

21. Lines 329. Do the DALY and health expenditures cited here refer to “smoking-attributable” estimates?

Response: Yes, we added “smoking-attributable” and “an associated health expenditure” to make this more explicit – line 330

22. Lines 340: “…is lower than our previous estimations [22]”. It is helpful to let the readers know what the previous estimates are.

Answer: This information has been provided and a reference has been added. The paragraph read now as follows:

"In this analysis, we found that the impact of a 50% increase in the price of a pack of cigarettes would be a reduction of 13,655 deaths over ten years, a value that is less than our previous estimates of 20,502 deaths prevented in ten years [49]. However, this previous analysis used data for 2015 when there was a higher smoking prevalence". lines 340-343
